# An Investigation of the Use of Traditional Chinese Medicine in Stroke Patients in Taiwan

**DOI:** 10.1155/2012/387164

**Published:** 2012-12-11

**Authors:** Chien-Chang Liao, Jaung-Geng Lin, Chin-Chuan Tsai, Hsin-Long Lane, Ta-Chen Su, Hwang-Huei Wang, Fung-Chang Sung, Ta-Liang Chen, Chun-Chuan Shih

**Affiliations:** ^1^Department of Anesthesiology, Taipei Medical University Hospital, Taipei 110, Taiwan; ^2^Management Office for Health Data, China Medical University Hospital, Taichung 404, Taiwan; ^3^Graduate Institute of Chinese Medicine, China Medical University, Taichung 404, Taiwan; ^4^School of Chinese Medicine for Post-Baccalaureate, I-Shou University, Kaohsiung City 84001, Taiwan; ^5^Department of Cardiology, National Taiwan University Hospital, Taipei 100, Taiwan; ^6^Graduate Institute of Integrated Medicine, College of Chinese Medicine, China Medical University, Taichung 404, Taiwan; ^7^Department of Public Health, China Medical University, Taichung 404, Taiwan; ^8^Chinese Medical Association, Taipei 100, Taiwan

## Abstract

*Background*. The use of complementary and alternative medicine in critical illness is increasing worldwide. This study investigates how traditional Chinese medicine (TCM) is used in stroke patients. *Methods*. Using Taiwan National Health Insurance reimbursement claims, we compared the annual use of TCM between stroke patients and general population, identifying 15,330 patients with a new onset of stroke in 2000–2009. The sociodemographic status and medical comorbidities between stroke patients receiving TCM services and those without using the service were compared. *Results*. The use of TCM was higher in stroke patients than in the general population, 27.9% versus 25.4% in 2000 and 32.7% versus 27.8% in 2009, respectively, and grew consistently from 2000 to 2009. Among stroke patients, women, younger patients, white-collar employees, higher-income residents, and those living in areas with more TCM physicians were more likely to use TCM. Stroke patients using rehabilitation services were more likely to have more TCM visits (OR = 2.28, 95% CI = 1.96–2.66) and higher expenditure on TCM (OR = 2.67, 95% CI = 2.29–3.12) compared with stroke patients without rehabilitation. *Conclusion*. TCM is popular and well accepted in Taiwan. Patients with stroke have a higher TCM utilization rate than people without stroke.

## 1. Introduction

Although the incidence of stroke is declining worldwide [1], it remains the leading cause of acquired disability in adults and the second leading cause of death in most areas [2, 3]. Risk factors, prevention strategy, and genetic biomarkers of stroke have been well studied [3–5]. Stroke patients commonly suffer from sequelae and complications such as dementia, depression, pneumonia, urinary tract infection, decubitus, fall (hip fracture), epilepsy, dysphagia, and constipation [6, 7]. Rehabilitation such as physical therapy may prevent sequelae after stroke or improve functional status [8]. Increasing numbers of stroke patients have sought alternative complementary and alternative medicine (CAM) or therapy to improve physical functions in recent years that is worthy of our attention [9–12].

Traditional Chinese medicine (TCM) is widely used in Taiwan [13–16] and other Asian countries [17–20]. Previous studies found that 10.4%, 28.4%, and 62.5% of the general population have used TCM services in the previous month, year, and six years in Taiwan, respectively [13, 16, 21]. After stroke chronic symptoms such as spasticity, changed muscle tone, motor neuron excitability, and ankle plantar flexor spasticity can improve after TCM treatment, particularly acupuncture [22–26]. Our previous studies also proved the medical effectiveness of acupuncture [27, 28].

To our knowledge, this is the first population-based study of characteristics of TCM use in stroke patients. This study used medical claims from Taiwan's National Health Insurance Research Database to evaluate patterns of TCM use in stroke patients.

## 2. Methods

### 2.1. Study Design and Sample

Taiwan's National Health Research Institutes has documented all the medical claims for insured beneficiaries since 1996 as the National Health Insurance Research Database for public access. With patient identification numbers scrambled, data files can be secured to protect patient privacy. Information available for this study included gender, birthday, disease codes, health care rendered, medicines prescribed, diagnoses for admissions and discharges, and medical institutions and physicians providing services. From a longitudinal cohort data set of a randomly selected one million insured subjects, we identified patients aged ≥20 years old with newly diagnosed stroke who were hospitalized in 2000–2009 as our eligible study subjects. In order to confirm that all stroke patients in our study were incident cases, only new-onset stroke cases were included in this study. The diagnosis of stroke was validated in previous studies [29]. Overall, 15,330 new-onset stroke patients were included in this study and followed to analyze their TCM use for one year. The outcome of this study is the rate of TCM use for people with the diagnosis of stroke in the first year. This study compared sociodemographic factors, coexisting medical conditions, and the characteristics of stroke between stroke patients with and without TCM use. The objective of this study is to investigate factors associated with the first-year use of TCM for people suffering from stroke.

### 2.2. Criteria and Definition

We defined stroke according to the International Code of Diseases, Ninth Edition, Clinical Modification (ICD-9-CM 430–438). Coexisting medical conditions included hypertension (ICD-9-CM 401–405), diabetes (ICD-9-CM 250), hyperlipidemia (ICD-9-CM 272.0–272.4), and myocardial infarction (ICD-9-CM 410 and 412). From individuals' health reimbursement claims, regular renal dialysis (including hemodialysis and/or peritoneal dialysis) was also considered a medical condition among stroke patients in this study. We classified the frequency of visits for TCM into quartiles. Stroke patients in the highest quartile of TCM visits were defined as high TCM users. Medical expenditure on TCM was also classified into quartiles. Stroke patients who had the highest quartile of TCM expenditure were considered high TCM expenditure patients.

As Taiwan has 359 townships and city districts, we calculated the population density (persons/km^2^) for each of these administrative units. Based on population density, these units were stratified into tertiles to designate areas of low, moderate, and high urbanization [14–16]. We calculated the density of TCM physicians (TCM physicians/10,000 persons) by using the number of TCM physicians per 10,000 residents for each of the administrative units. The first, second, and third tertiles were considered as areas with low, moderate, and high physician density, respectively [14, 16].

### 2.3. Statistical Analysis

The proportions of stroke patients using TCM services were calculated annually from 2000–2009 and compared with patients without stroke to observe trends in TCM use. Patients with stroke were further divided into two groups, those who did and did not use TCM. The sociodemographic status and comorbidities between these two groups were compared and examined using chi-square tests and analysis of variance. Univariate and multivariate logistic regression analyses were performed to calculate crude and adjusted odds ratios (ORs) and 95% confidence intervals (CIs) measuring relationships between TCM use and associated factors for stroke patients. These factors included age, sex, occupation, low-income status, urbanization, density of TCM physicians in area, history of diseases, use of rehabilitation, types of stroke, and in-hospital characteristics. All analyses were performed using Statistical Analysis Software version 9.1 (SAS Institute Inc., Cary, North Carolina, USA). A two-sided probability value of <0.05 was considered significant.

### 2.4. Ethical Approval

This study was conducted in accordance with the Helsinki Declaration. To protect personal privacy, the electronic database was decoded for research with patient identification scrambled. According to National Health Research Institutes regulations, informed consent is not required because patient identification has been decoded. This study was evaluated and approved by Taiwan's National Health Research Institutes.

## 3. Results

**Figure 1:**
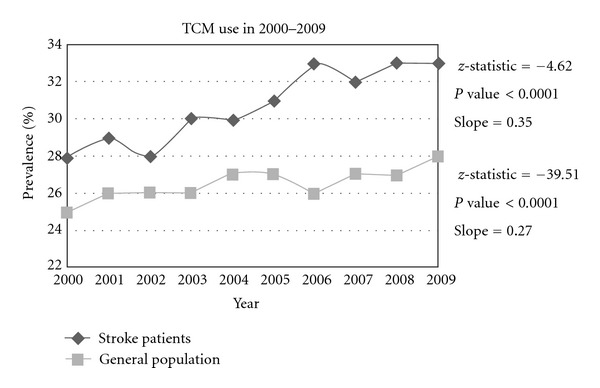
Use of traditional Chinese medicine services by patients with stroke and general population, 2000–2009 (use Cochran-Amitage Trend Test).

The prevalence of TCM use among stroke patients increased from 24% in 2000 to 32% in 2009 (*P* < 0.0001) (Figure 1). TCM use was higher in stroke patients than in general population annually from 2000–2009 (*P* < 0.0001) (Table 1). Compared with nonusers, higher proportions of TCM users were females (43.6% versus 41.3%, *P* = 0.007), younger patients (36.8% versus 26.6%, *P* < 0.0001), white-collar employees (38.7% versus 31.9%, *P* < 0.0001), residents living in highly urbanized areas (59.4% versus 54.6%, *P* < 0.0001), and areas with more TCM physicians (28.3% versus 23.7%, *P* < 0.0001). Low-income stroke patients were less likely to be TCM users (0.7% versus 1.2%, *P* = 0.004). TCM users were more likely to use other rehabilitation services than non-TCM users (43.9% versus 28.1%, *P* < 0.0001). TCM users also experienced more comorbidities with hypertension (64.4% versus 59.8%, *P* < 0.0001) and hyperlipidemia (12.2% versus 9.2%, *P* < 0.0001) but less renal dialysis (0.8% versus 2.1%, *P* < 0.0001).

The multivariate logistic regression analysis showed ORs of factors associated with higher TCM uses in stroke patients (Table 2), including women (OR = 1.16, 95%  CI = 1.08–1.25), younger patients (OR = 3.35, 95%  CI = 2.36–4.76), white-collar employees (OR = 1.38, 95% CI = 1.22–1.55), people without low income (OR = 1.55, 95% CI = 1.04–2.32), areas with more TCM physicians (OR = 1.34, 95%  CI = 1.20–1.49), use of other types of rehabilitation (OR = 2.15, 95%  CI = 1.99–2.32), and patients with comorbidities of hypertension (OR = 1.29, 95%  CI = 1.20–1.39) and hyperlipidemia (OR = 1.29, 95%  CI = 1.15–1.44) and without renal dialysis (OR = 2.79, 95%  CI = 1.97–3.95). Shorter hospitalization was a factor associated with TCM use (OR = 1.14, 95%  CI = 1.05–1.24).

The average of TCM visits in stroke patients was higher in young adults than in older people (9.8 ± 16.4 versus 7.8 ± 10.6, *P* = 0.0009). Stroke patients who were white-collar employees, who lived in highly urbanized areas and areas with more TCM physicians, or who used rehabilitation services made more TCM visits (Table 3). Patients who had more visits for TCM care also had higher expenditure for TCM use.

## 4. Discussion

This study compared trends in TCM use by patients with stroke and general population to investigate clinical factors associated with TCM use in stroke patients. TCM use was consistently higher among stroke patients than in general patients from 2000 to 2009. TCM use in patients with stroke is highly correlated with sociodemographic characteristics. 

These data challenge the biomedical profession's previous assumption that the biomedical system defines our society's health care practices. To the best of our knowledge, our study is the first population-based study to analyze the use of TCM and to study its association with stroke patients.

The increasing use of CAM [9–11, 30] or TCM [13–20, 30–33] has been reported in both Asian and Western countries. Consistent with previous reports indicating increased use of TCM in general populations [13–16], the present study found that the prevalence of TCM use in stroke patients increased from 24% in 2000 to 32% in 2009. TCM is considered a subdivision of CAM in Western countries, with acupuncture treatment increasingly used in Western societies [34, 35], particularly for stroke patients [22, 23, 25, 36–42]. Evidence-based studies have shown TCM's beneficial effects in addressing physical and mental illnesses in stroke patients [22–26, 36–38].

Demographic factors such as age and sex are associated with patient choice of TCM [13, 14, 16, 18, 19, 21, 31]. Young and middle-aged adults were more likely than older people to use TCM; this trend also extends to foreign white-collar workers in Taiwan [42]. Many studies demonstrate that TCM herbal medicine is helpful in maintaining regular menstruation [43]; this may help to explain why females were more likely to adopt TCM than males. A previous study suggested that young people are more likely to actively seek ways to improve their well-being and relieve disease symptoms [37]. The similar association between age and TCM use was also reported in previous studies with different scenarios [13, 14, 16, 31]. It is thus reasonable that younger stroke patients had a higher tendency to use TCM and to represent higher TCM expenditure than older patients in this study.

Economic growth is a major determinant of physician supply and medical utilization [44]. TCM has become an increasingly popular form of medicine in Taiwan, particularly since the universal coverage for such services by the National Health Insurance. The increasing use of TCM is interacting with the growth in the number of TCM physicians in Taiwan [45]. Residents in urban areas have more access to TCM services than rural residents because more TCM physicians practice in cities than in rural areas [14, 16]. Thus we found that high urbanization was a factor associated with TCM use.

Among the stroke patients, hypertension, hyperlipidemia, diabetes, uremia, and myocardial infarction were common coexisting medical conditions considered as comorbidities in this study. We found that patients with comorbidities of hypertension or hyperlipidemia and patients without renal dialysis were more likely to use TCM care. The beneficial effects of TCM treatment on hypertension and hyperlipidemia have been reported in previous studies [46, 47]. Patients with end-stage renal disease receiving dialysis two or three times weekly might have less opportunity to use TCM. In contrast, stroke patients receiving TCM treatment have shorter hospitalization. Our further analysis showed patients with hypertension had longer hospitalization (not shown in the tables). In addition, we found that stroke patients using TCM were more likely to use other types of physical treatment and rehabilitation simultaneously. Further analysis revealed that these patients faced lower risk of recurrent stroke (not shown in tables). The frequency of TCM visits and related expenditure were higher in stroke patients receiving physical treatment and rehabilitation. Our previous study [8] discussed how stroke patients who accessed regular rehabilitation services may have better knowledge, attitudes, and practices regarding disease prevention and how this population may embrace pluralistic attitudes about health care [13]. Healthy lifestyles were also a characteristic of TCM users [14, 16].

While previous studies provided by self-reported information collected through interviews, the current study used the Taiwan National Health Insurance claims data that may avoid recall bias. In addition, our study used multivariate logistic regression analysis to study factors associated with TCM use in stroke patients after adjustment for covariates. To the best of our knowledge, this study is the first population-based study showing the utilization of TCM and its associated factors in stroke patients.

This study has some limitations. First, we used retrospective medical claims data from health insurance that lacked detailed patient information on lifestyle as well as physical, psychiatric, and laboratory examinations. We were unable to differentiate whether these factors were causally related with TCM use. Second, our study used ICD-9-CM codes claimed by physicians for the diagnosis of stroke without clarifying the severity of disease. We supposed that severe stroke patients may have had less opportunity to use TCM because of their activity limitations and longer hospital stays. Third, information on folk therapy, which has been reported as a predictor for TCM use, was not available in the National Health Insurance Research Database [13, 14, 16]. Finally, this study was based on cross-sectional analyses of TCM use in stroke patients. The beneficial effects of TCM in stroke patients require further cohort studies.

In conclusion, the application of TCM in stroke patients is well accepted and increasing in Taiwan. Stroke patients use TCM more often than people without stroke, and this is associated with sociodemographic factors and clinical characteristics. This study may provide some clinical implications of CAM in these populations that are useful for health professionals worldwide.

## Figures and Tables

**Table 1:** Comparisons of sociodemographic characteristics, comorbidities, and other medical conditions between stroke patients using traditional Chinese medicine or not.

	TCM use				*P* value
	No (*N* = 10605)		Yes (*N* = 4725)	
Sex	*n*	(%)	*n*	(%)	0.007
Women	4376	(41.3)	2059	(43.6)	
Men	6229	(58.7)	2666	(56.4)	
Age, years					<0.0001
20–29	73	(0.7)	65	(1.4)	
30–59	2746	(25.9)	1675	(35.4)	
60–69	2514	(23.7)	1309	(27.7)	
≥70	5272	(49.7)	1676	(35.5)	
Mean ± SD	68.0 ± 13.3		63.7 ± 13.1		<0.0001
Occupation, white collar	3382	(31.9)	1828	(38.7)	<0.0001
Low income	126	(1.2)	32	(0.7)	0.004
Urbanization					<0.0001
Low	754	(7.1)	270	(5.7)	
Moderate	4063	(38.3)	1648	(34.9)	
High	5788	(54.6)	2807	(59.4)	
Density of TCM physicians					<0.0001
Low	2838	(26.8)	1031	(21.8)	
Moderate	5256	(49.6)	2358	(49.9)	
High	2511	(23.7)	1336	(28.3)	
Rehabilitation	2982	(28.1)	2072	(43.9)	<0.0001
Hypertension	6343	(59.8)	3044	(64.4)	<0.0001
Diabetes mellitus	3102	(29.3)	1385	(29.3)	0.94
Hyperlipidemia	972	(9.2)	578	(12.2)	<0.0001
Myocardial infarction	241	(2.3)	98	(2.1)	0.44
Renal dialysis	220	(2.1)	39	(0.8)	<0.0001
Ischemic stroke	6481	(61.1)	2839	(60.1)	0.29
Length of stay, <10 day	6797	(64.1)	3054	(64.6)	0.52
Mean ± SD	11.8 ± 18.9		11.8 ± 14.8		0.93

SD: standard deviation; TCM: traditional Chinese medicine.

**Table 2:** Factors associated with use of traditional Chinese medicine among stroke patients in multivariate logistic regression model.

	Number	TCM use*
		Adjusted OR (95% CI)
Sex		
Women	6435	1.16 (1.08–1.25)
Men	8895	1.00 (reference)
Age, years		
20–29	138	3.35 (2.36–4.76)
30–59	4421	1.94 (1.77–2.12)
60–69	3823	1.60 (1.46–1.75)
≥70	6948	1.00 (reference)
Occupation		
White collar	5210	1.38 (1.22–1.55)
Blue collar	7763	1.21 (1.08–1.36)
Others	2357	1.00 (reference)
Urbanization		
Low	1024	1.00 (reference)
Moderate	5711	1.04 (0.89–1.22)
High	8595	1.09 (0.93–1.29)
Density of TCM physicians		
Low	3869	1.00 (reference)
Moderate	7614	1.12 (1.01–1.24)
High	3847	1.34 (1.20–1.49)
Without low-income status	15172	1.55 (1.04–2.32)
Used rehabilitation therapy	5054	2.15 (1.99–2.32)
Without diabetes mellitus	10843	1.02 (0.94–1.10)
Hypertension	9387	1.29 (1.20–1.39)
Hyperlipidemia	1550	1.29 (1.15–1.44)
Without myocardial infarction	14991	1.03 (0.81–1.32)
Without renal dialysis	15071	2.79 (1.97–3.95)
Length of stay, <10 days	9851	1.14 (1.05–1.24)

OR: odds ratio; CI: confidence interval; TCM: traditional Chinese medicine.

*Hosmer-Lemeshow goodness of fit, *P* = 0.16; c-statistic = 0.65.

**Table 3:** Stratification analyses of sociodemographic factors and medical conditions on averaged frequency of visits and expenditure on traditional Chinese medicine among stroke patients (*N* = 4725).

	TCM visits		TCM expenditure	
	Mean ± SD	*P* value	Mean ± SD	*P* value
Sex				
Women	8.1 ± 10.7	0.13	192 ± 298	0.02
Men	8.6 ± 11.0		212 ± 314	
Age, years				
20–29	9.8 ± 16.4	0.0009	250 ± 514	0.0003
30–59	9.2 ± 11.5		228 ± 332	
60–69	8.1 ± 9.9		193 ± 276	
≥70	7.8 ± 10.6		186 ± 294	
Occupation				
White collar	9.3 ± 11.6	<0.0001	231 ± 334	<0.0001
Blue collar	7.6 ± 10.0		179 ± 272	
Others	8.7 ± 11.8		218 ± 344	
Urbanization				
Low	7.3 ± 10.2	0.002	179 ± 288	0.0003
Moderate	7.8 ± 10.2		183 ± 286	
High	8.9 ± 11.3		218 ± 321	
Density of TCM physicians				
Low	7.5 ± 9.7	0.002	170 ± 260	0.0002
Moderate	8.5 ± 11.0		210 ± 317	
High	9.1 ± 11.4		219 ± 323	
Low income				
No	8.4 ± 10.8	0.49	203 ± 307	0.50
Yes	10.3 ± 15.0		254 ± 420	
Rehabilitation				
No	6.5 ± 8.7	<0.0001	138 ± 204	<0.0001
Yes	10.8 ± 12.8		288 ± 387	
Diabetes mellitus				
No	8.5 ± 11.0	0.29	205 ± 308	0.56
Yes	8.2 ± 10.5		200 ± 306	
Hypertension				
No	8.7 ± 11.7	0.16	216 ± 343	0.06
Yes	8.2 ± 10.4		197 ± 286	
Hyperlipidemia				
No	8.4 ± 10.8	0.58	203 ± 307	0.84
Yes	8.6 ± 11.1		206 ± 312	
Myocardial infarction				
No	8.4 ± 10.9	0.79	204 ± 308	0.83
Yes	8.7 ± 10.2		210 ± 289	
Renal dialysis				
No	8.4 ± 10.9	0.15	204 ± 308	0.31
Yes	6.8 ± 6.8		168 ± 215	

SD: standard deviation; TCM: traditional Chinese medicine.
